# The Skull Vibration-Induced Nystagmus Test of Vestibular Function—A Review

**DOI:** 10.3389/fneur.2017.00041

**Published:** 2017-03-09

**Authors:** Georges Dumas, Ian S. Curthoys, Alexis Lion, Philippe Perrin, Sébastien Schmerber

**Affiliations:** ^1^Department of Oto-Rhino-Laryngology, Head and Neck Surgery, University Hospital, Grenoble, France; ^2^EA 3450 DevAH, Development, Adaptation and Disadvantage, Faculty of Medicine and UFR STAPS, University of Lorraine, Villers-lès-Nancy, France; ^3^Vestibular Research Laboratory, School of Psychology, the University of Sydney, Sydney, NSW, Australia; ^4^Sports Medicine Research Laboratory, Luxembourg Institute of Health, Strassen, Luxembourg; ^5^Department of Paediatric Oto-Rhino-Laryngology, University Hospital of Nancy, Vandoeuvre-lès-Nancy, France; ^6^INSERM UMR 2015, Grenoble, France

**Keywords:** skull vibration, nystagmus, vertigo, high frequencies, vestibular disease

## Abstract

A 100-Hz bone-conducted vibration applied to either mastoid induces instantaneously a predominantly horizontal nystagmus, with quick phases beating away from the affected side in patients with a unilateral vestibular loss (UVL). The same stimulus in healthy asymptomatic subjects has little or no effect. This is skull vibration-induced nystagmus (SVIN), and it is a useful, simple, non-invasive, robust indicator of asymmetry of vestibular function and the side of the vestibular loss. The nystagmus is precisely stimulus-locked: it starts with stimulation onset and stops at stimulation offset, with no post-stimulation reversal. It is sustained during long stimulus durations; it is reproducible; it beats in the same direction irrespective of which mastoid is stimulated; it shows little or no habituation; and it is permanent—even well-compensated UVL patients show SVIN. A SVIN is observed under Frenzel goggles or videonystagmoscopy and recorded under videonystagmography in absence of visual-fixation and strong sedative drugs. Stimulus frequency, location, and intensity modify the results, and a large variability in skull morphology between people can modify the stimulus. SVIN to 100 Hz mastoid stimulation is a robust response. We describe the optimum method of stimulation on the basis of the literature data and testing more than 18,500 patients. Recent neural evidence clarifies which vestibular receptors are stimulated, how they cause the nystagmus, and why the same vibration in patients with semicircular canal dehiscence (SCD) causes a nystagmus beating toward the affected ear. This review focuses not only on the optimal parameters of the stimulus and response of UVL and SCD patients but also shows how other vestibular dysfunctions affect SVIN. We conclude that the presence of SVIN is a useful indicator of the asymmetry of vestibular function between the two ears, but in order to identify which is the affected ear, other information and careful clinical judgment are needed.

## Introduction—Historical Background

Von-Bekesy in 1935 (1) reported that vibration applied to the skull induced reflexes and motion illusions which he attributed to stimulation of vestibular receptors. Lucke in 1973 (2) first described how 100 Hz mastoid vibration-induced nystagmus (VIN) in a patient with a unilateral vestibular loss (UVL), and in 1999 that result was confirmed and extended by Hamann and Schuster (3) and Dumas et al. (4, 5). Dumas et al. described a systematic clinical analysis of the skull vibration-induced nystagmus (SVIN) in patients after total (tUVL) [after surgery for vestibular schwannoma (VS)] or partial (pUVL) UVL and reported more recently SVIN in superior semicircular canal dehiscence (SCD) patients (6, 7).

Shortly after Lucke’s observation, Young et al. (8) reported that squirrel monkey primary afferents from semicircular canals (SCC) and otoliths were activated by bone-conducted vibration (BCV). That report presaged the likely explanation of SVIN and the neural basis of SVIN is considered below.

Vibration-induced nystagmus has been described in various inner ear diseases (Table 1); most publications address cranial BCV stimulations (2–7, 9–25, 27–29, 41–43, 45) but others deal with cervical and cranial vibrations (26, 30, 31) and a few with cervical stimulations only (32–34, 40, 44). This review is only mainly restricted to BCV with cranium stimulations which are now more clearly documented by physiology (35–39). We propose for clarity to term it the skull vibration-induced nystagmus test (SVINT).

**Table 1:** **Synoptic table of results in literature**.

Reference	Level of evidence	Study design	Sample size (*n*)	Pathology	Record	Stimulus location	Stimulus frequency Hz (amplitude mm)	Main contribution, comments
Lücke 1973 (2)	3	RCS	65	Unilateral vestibular loss (UVL) patients, central patients	Frenzel	Face cranium vertex necknape	100	First incidental observation of a vibration-induced nystagmus (VIN) in a UVL patient
Lackner and Graybiel 1974 (11)	2	PCS	6	Normal subjects	Frenzel	Face, mastoids, cervical	40–280 optimal 120–180	Vibrations induce postural, visual illusions, rare VIN in normal subjects
Yagi and Ohyama 1996 (32)	3	PCS	11	UVL	VNG3D	Dorsal neck muscles	115 (1 mm)	Vibrations induce in UVL compensated patients a VIN (Hor and Vert components) related to vestibular decompensation
Strupp et al. 1998 (33)	2	PCS	25	VN	VNG, SVSA	Neck muscles	100	Somatosensory substitution of vestibular function in UVL patients
			25	Controls				
Popov et al. 1999 (40)	2	PCS	4	UVL	Scleral, coils, visual illusions	Neck vibration	90 (0.5 mm)	Propriogyral illusion secondary to vibration-induced eye movement (COR)
			5	Controls				
Hamann and Schuster 1999 (3)	3	RCS	60	Peripheral UVL benign positional paroxystic vertigo	VNS VNG2D	Mastoid	60, 100	In UVL, a lesionnal VIN is observed in peripheral diseases and seldom in BPPV and in central patients. Optimal stim 60 Hz
			40	BSL				
Dumas et al. 1999 (4)	3	RCS	80	UVL: TUVL (TA, VNT) PUVL (MD, VN, VS)	VNS, VNG3D	Mastoid, vertex	100 (0.2 mm)	VIN: 3 components in TUVL. VIN characteristics, technical conditions, sensitivity, specificity
			10	BSL				
			100	Controls				
Dumas et al. 2000 (5)	3	RCS	46	UVL	VNS, VNG3D	Mastoid, vertex	20–150 (0.2 mm)	VIN SPV amplitude; location and frequency stimulus optimization. A vestibular Weber test
			105	Controls				
Karlberg et al. 2003 (13)	3	PCS	18	UVL (VN, VNT)	Scleral Coils, SVH	Mastoid, posterior neck	92 (0.6 mm)	SVH shift is explained by vibration-induced ocular torsion whose magnitude is related to the extent of UVL deficit
Ohki et al. 2003 (12)	3	RCS	100	UVL (VN, MD, VS)	VNG	Mastoid, forehead	100	In UVL patients VIN is correlated with CaT hypofunction
Nuti and Mandala 2005 (21)	3	RCS	28	VN	VNG	Mastoid	60–120	Sensitivity 75%, specificity 100% VIN beats usually toward the intact side
			25	Controls				
Magnusson et al. 2006 (31)	2	PCS	10	Normal subjects	Posture	Mastoid, neck	85 (1 mm) 55 (0.4 mm)	Cervical muscle afferents play a dominant role over vestibular afferents during bilateral vibration of the neck
Dumas et al. 2007 (10)	3	RCS	4,800	TUVL, PUVL, brainstem lesion	VNS, VNG	Mastoid, vertex	100 (1 mm)	VIN is observed in 98% TUVL,75% PUVL, 34% BSL
Hong et al. 2007 (22)	3	RCS	52	MD Unilat	VNS, VNG, head-shaking-nystagmus (HSN), CaT	Mastoid	100	VIN is usually correlated with CaT hypofunction. VIN beats frequently ipsilaterally toward MD side
White et al. 2007 (41)	3	RCS	8	SCD	VNS, VNG 2D	Mastoid, vertex, suboccip.	100	Vibrations induce a torsional VIN beating toward the SCD and down beating suggesting the stimulation of the dehiscent SSCC
Dumas et al. 2008 (26)	3	RCS	131	TUVL (TA, VNT)	VNS, VNG 2D 3D	Mastoid, vertex (cervical)	100 (1 mm)	VIN: 3 components (H,V,T), SVINT: a bilateral stimulation, sensitivity 98%, specificity 94%, SPV:10.7°/s; SD = 7.5, VIN is always beating toward the intact side
			95	Controls				
Manzari et al. 2008 (42)	3	RCS	16	SCD	VNG3D	Mastoid	100	Vibrations induce a VIN with a torsional component beating toward the lesion side
Park et al. 2008 (23)	3	RCS	19	VN	VNG	Mastoid	100	Clinical significance of VIN
			22	Controls				
Park et al. 2010 (24)	2	PCS	26	VN	VNG	Mastoid	100	VIN clinical significance, reliability
Aw et al. 2011 (43)	3	RCS	17	SCD	Scleral coils	Mastoid	500	Eye slow torsional component ViVOR is directed toward the intact side: vibrations stimulate the anterior dehiscent canal
Dumas et al. 2011 (27)	3	RCS	99	PUVL (VN, VS, MD, CL)	VNG 2D	Mastoid, vertex	30, 60, 100 (1 mm)	Sensitivity 75%. VIN beats toward safe side in 91%. skull vibration-induced nystagmus test complements CaT, HST in vestibular multifrequential analysis
Kawase 2011 (44)	3	RCS	14	7 pre-surgical VS, 7 post-surgical VS	VNG, SVV	Neck muscles	110	Ipsilat. vibrations increase SVV deviation, VIN is correlated to SVV alteration, VIN is not modified by the side of the stimulation
Koo et al. 2011 (19)	3	RCS	74	VS	VNG	Mastoid	100	Comparison of sensitivity of VIN and other vestibular tests in the YAW axis in VN. VIN is observed in 86% of cases in correlation with CaT Hypofunction. VIN beats toward the intact side in 98% VIN is observed in 86% of cases in correlation with CaT Hypofunction. VIN beats toward the intact side in 98%
			24	Controls	HST CaT			
Dumas et al. 2013 (30)	2	RCS	9	Profound compensated long-standing UVL	VNG 2D, posturog	Mastoid, vertex (cervical)	100	VIN beats toward the intact side in 100% of cases, No measurable postural changes in EC condition in long standing compensated severe UVL patients
			12	Control				
Xie et al. 2013 (20)	3	RCS	112	UVL	VNG, HST CaT	Mastoids	100	VIN is observed in 91% of peripheral UVL. It is more frequent and important when CaT canal paresis augments. VIN usually beats toward the healthy side except in MD VIN specificity is 100%
			30	Controls				
Dumas et al. 2014 (7)	3	RCS	17	SCD (unilateral)	VNG 3D, cVEMP, CaT, VHIT	Mastoid, vertex	60,100 (1 mm)	In Unilat SCD, a VIN is observed in 86% cases. Horizotal and Torsional components beat toward lesion side. The VIN vert. component is most often up beating. Higher responses are obtained on vertex location
			12	Control				
Park et al. 2014 (25)	3	RCS	11	SCD		Mastoid	100	VIN horizontal component beats toward the lesion side
Lee et al. 2015 (45)	3	RCS	87	MD	VNG	Mastoid	100	In MD, VIN and HSN are not always in the same direction
						Front		
Dumas et al. 2016 (46)	2	PCS	11	Normal subjects	Piezoelectric sensor	Mastoid; vertex; neck	100	Vibration transfer is more efficient from one mastoid to the other one

*RCS, Retrospective Clinical Study; PCS; Prospective Clinical Study; TUVL, total unilateral vestibular lesion; PUVL, partial unilateral vestibular lesion; VN, vestibular neuritis; MD, Meniere’s disease; VS, vestibular Schwannoma; CL, chemical labyrinthectomy (Gentamicin); SCD, superior semicircular canal dehiscence; TA, translabyrinthine approach (for VS surgery); VNT, vestibular neurotomy; BSL, brainstem lesion; VNG, videonystagmography; VNS, videonystagmoscopy; 3D, 3-dimensional study of the nystagmus; 2D, 2-dimensional study; SSC, scleral searching coils; SVV, subjective visual vertical; SVH, subjective visual horizontal; SVSA, subjective visual straight ahead; CaT, caloric test; cVEMP, cervical evoked myogenic potentials; VIN, vibration induced nystagmus; HSN, head shaking nystagmus; COR, cervico–ocular reflex; EC, eye closed*.

## Methods—Practical Conditions

### Test Procedure

**Figure 1:**
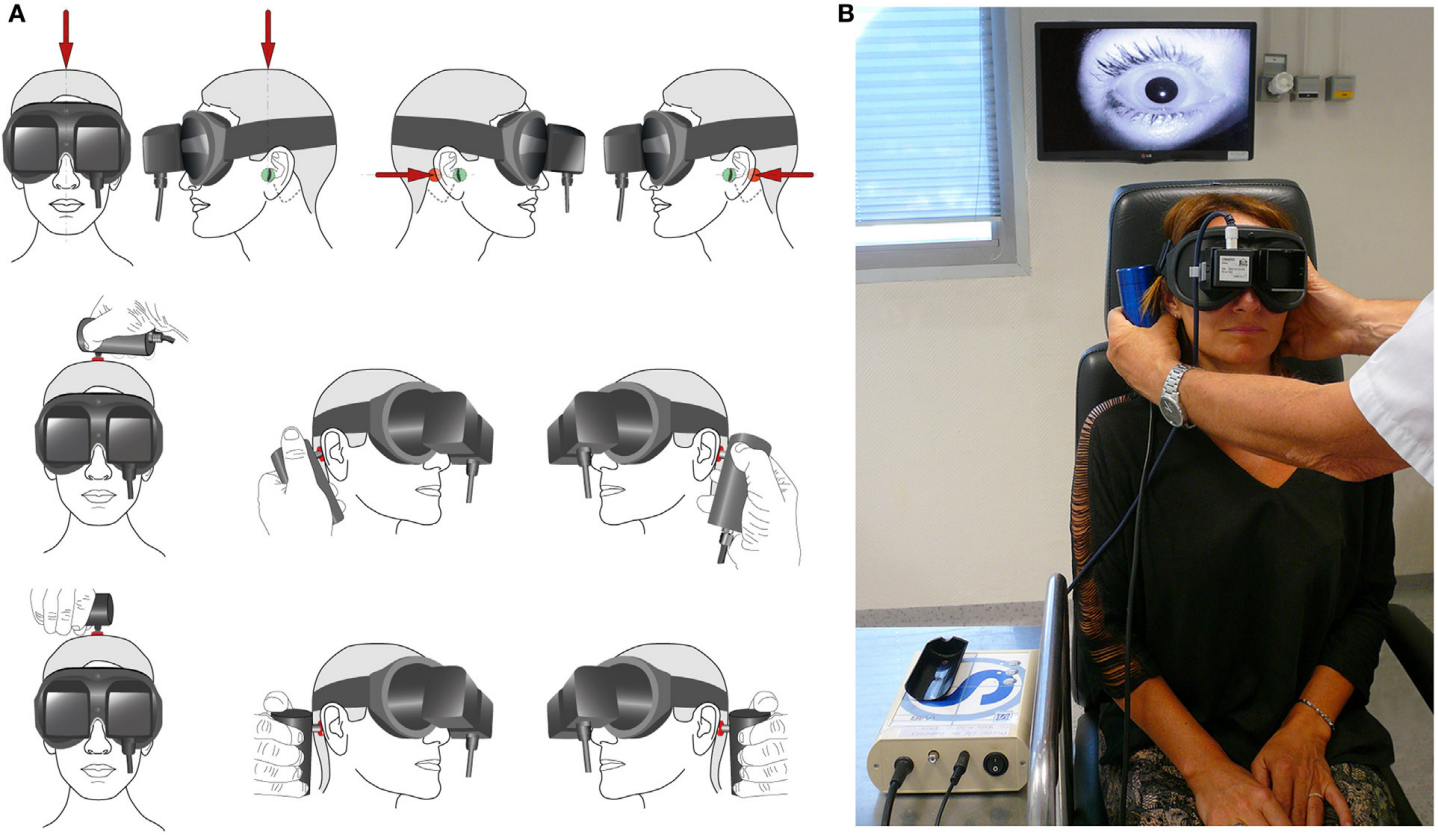
**Skull vibration-induced nystagmus test technique in clinical practice**. **(A)** Principle of stimulation: the examiner can face the subject, as in the first example. The vibrator cylindrical contact is applied perpendicularly to the designated surface (red spot) with a pressure of about 10 N or 1 kg on the vertex or each mastoid process [level to the external acoustic meatus (Green spot)]. The examiner uses the other hand to maintain and immobilize the subject’s head. The same type of stimulation can be performed with the examiner behind the subject (second example situation). Stimulation must avoid the mastoid tip to prevent from muscular vibration radiation and proprioceptive involvement. **(B)** Mastoid stimulation; examiner in front of the subject; the other hand immobilizes the head. 3F Synapsys stimulator (France). Videonystagmoscopic recording (Collin ORL, France).

The examiner performs stimulation either by standing in front of (or behind) the patient to use his dominant hand for more reproducibility (46, 47). The vibrator must be firmly held and applied perpendicularly to the skin over the mastoid process, posteriorly to the auricle, at the level of the external acoustic meatus (Figure 1). Stimulation applied on the tip of the mastoid process must be avoided, as it can induce activation of proprioceptive afferents from the trapezius and sternocleidomastoid muscles (47). A pressure of about 10 N is applied. It is recommended that three stimulation trials of each mastoid be given using 100 Hz with each stimulus lasting about 5–10 s. Eye movements can be visualized either under Frenzel goggles or, preferably, observed using video procedures such as videonystagmoscopy or recorded under videonystagmography 2D or 3D. Testing must be done in complete absence of any visual fixation of either eye (47). The average slow-phase velocity (SPV) of SVIN after tUVL is 10.83°/s (SD = 6.82; *n* = 45) (10, 26). The simplest procedure is to use only mastoid stimulation as described above (3, 12, 23, 24); one may use also vertex stimulation (4, 26, 29). The technical and practical conditions of the test are presented in Figure 1.

### Stimulation

Different vibrators are available [VVIB 3F or VVIB 100 Hz (Synapsys, France) or ISV 1 or IP 500 (Amplifon, France) or VVSED 500 (Euro Clinic, Italy) or NC 70209 (North Coast Medical, USA)] (Figure 1). The vibrator should preferably have a circular contact surface 20 mm in diameter covered with a thin felt or thin rubber.

**Figure 2:**
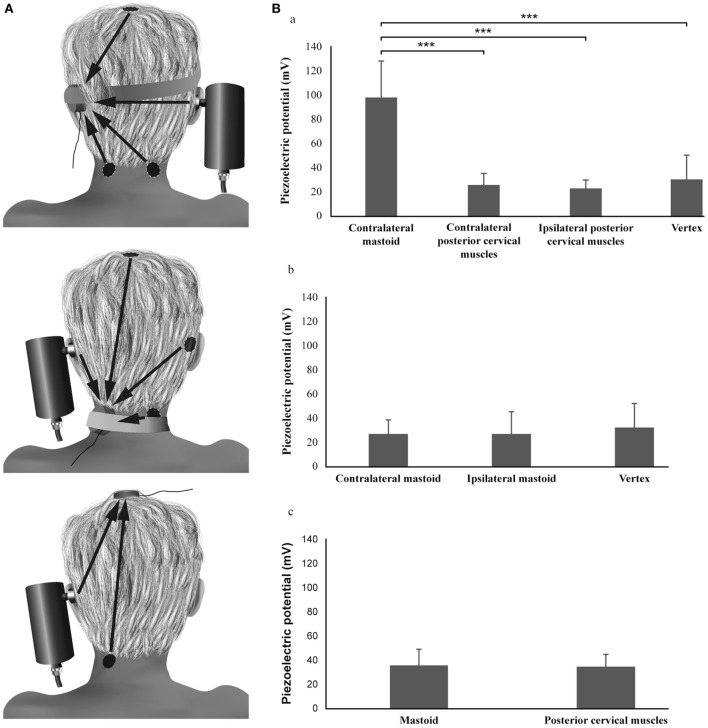
**Topographic optimization analysis of the skull vibration-induced nystagmus test**. **(A)** Procedure. **(B)** Results: the piezoelectric potentials (millivolts) recorded on the mastoid are significantly different according to the location of the stimulation (Friedman test, *P* < 0.001): the values obtained during the vibratory stimulation of the contralateral mastoid are higher than those obtained after vertex or ipsilateral and contralateral posterior cervical muscle vibrations (Wilcoxon tests, *P* < 0.001). No difference is observed between vertex and posterior cervical muscle stimulation locations (Wilcoxon tests, *P* > 0.05). The piezoelectric potentials recorded on the vertex or the posterior cervical muscles are not different according to the location of the stimulation (Friedman test and Wilcoxon test, *P* > 0.05).

Pressure and acceleration measures have shown that for optimum mastoid stimulation the force should be around 10 N or 1 kg (46). For such mastoid stimulation the spread or radiation of vibration to neck muscles is small (46). Figure 2 shows the SVINT topographic optimization using piezoelectric sensors. Vibration applied to one mastoid is very efficiently transmitted to the opposite mastoid for frequencies under 0.25 kHz (48–54); conversely vertex and cervical stimulations are less efficient for vibrations energy transfer to mastoid (close to the vestibule end organ). A small part of vibrations radiates to cervical region and *vice versa*.

### Stimulus Location

For most vestibular pathologies, mastoid stimulation elicits higher SVIN SPV than vertex or cervical stimulation (4, 5, 9, 10, 19–24, 26). Mastoid stimulation will predominantly stimulate labyrinthine receptors in both labyrinths, with only small stimulation of cervical muscle proprioceptors (46). For the cranial midline location, the comparison of the SVIN SPV measured at the frontal location (Fz), vertex (Vx), bregma, occipital, and sub-occipital locations at 100 and 60 Hz in 15 UVL patients did not show significant differences (46). BCV applied to the frontal location is considerably less efficient than mastoid vibration in eliciting SVIN (26) and for vibration transfer to the promontory (54–56).

However, in cases of SCD or other pathologies associated with a third window, vertex stimulation is more efficient than mastoid stimulation (7). This may occur because the pressure transmission from cerebrospinal fluid (*via* middle temporal fossa fistula) is enhanced in this condition (50, 57, 58).

### Stimulus Frequency

In measures on patients with tUVL, a large range of frequencies (40–150 Hz) have been shown to induce SVIN. Stimulation at 20 Hz is not effective, and progressively stronger responses are obtained for stimuli between 60 to 120 Hz (28), with around 100 Hz being optimal. In clinical practice, the frequency of stimulation used by Karlberg et al. (13) was 92 Hz, Lackner and Graybiel (11) was 120 Hz, and Magnusson et al. (31) was 85 Hz, and it was 100 Hz for the following: Manzari et al. (42), Ohki et al. (12), Park et al. (24), Koo et al. (19), Xie at al (20), and Dumas et al. (27).

In patients with pUVL, similar results are observed but with significant smaller slow-phase eye velocities (SPV) of SVIN. SVIN SPV were significantly higher at 100 Hz and 60 Hz than at 30 Hz (27). Recently our group has shown SPV was optimal at 100 Hz by testing SVIN frequencies from 10 to 700 Hz delivered by a Bruel & Kjaer Minishaker 4810 (Naerum, Denmark) in 15 common and severe UVL patients. No responses were observed at 10 or 500 Hz.

## Results – Main Leads for Interpretation (Box 1)

Box 1 **Main clinical outcomes.**

Skull vibration-induced nystagmus (SVIN) optimal frequency stimulation is 100 Hz and shows a primarily horizontal component; the best location is the mastoid process in most peripheral diseases except in SCD and other labyrinthine pathologies associated with a third window (higher responses are obtained on vertex). Both labyrinths are concomitantly stimulated, and a SVIN beating away from the lesion side is the result of the stimulation of the intact side in tUVL. In partial unilateral vestibular lesions (pUVL), a SVIN beating toward the intact side is usually obtained on mastoid process stimulation. But in SCD, SVIN beats toward the lesion side. The SVIN SPV is correlated in tUVL with the total caloric efficiency on the healthy ear. No responses are observed in bilateral areflexia or symmetrical hypofunctions. The SVIN is definitive and not modified at repetitive controls and long lasting re-tests. The sensitivity is 98% in tUVL and the specificity 94% in normal subjects. In pUVL, sensitivity is 75% and SVIN beats toward the intact side in 91% of cases. No significant alteration of the vestibulo-spinal reflex analyzed with posturography is observed in chronic compensated unilateral vestibular lesion (UVL) in eye-closed condition. SVIN Test is more sensitive to reveal peripheral than central neurological diseases. A 100 Hz BCV stimulates both canal and otolith structures in animals but SVIN in clinic is more relevant and is a good marker for canal lesions which it is well correlated to.

**Figure 3:**
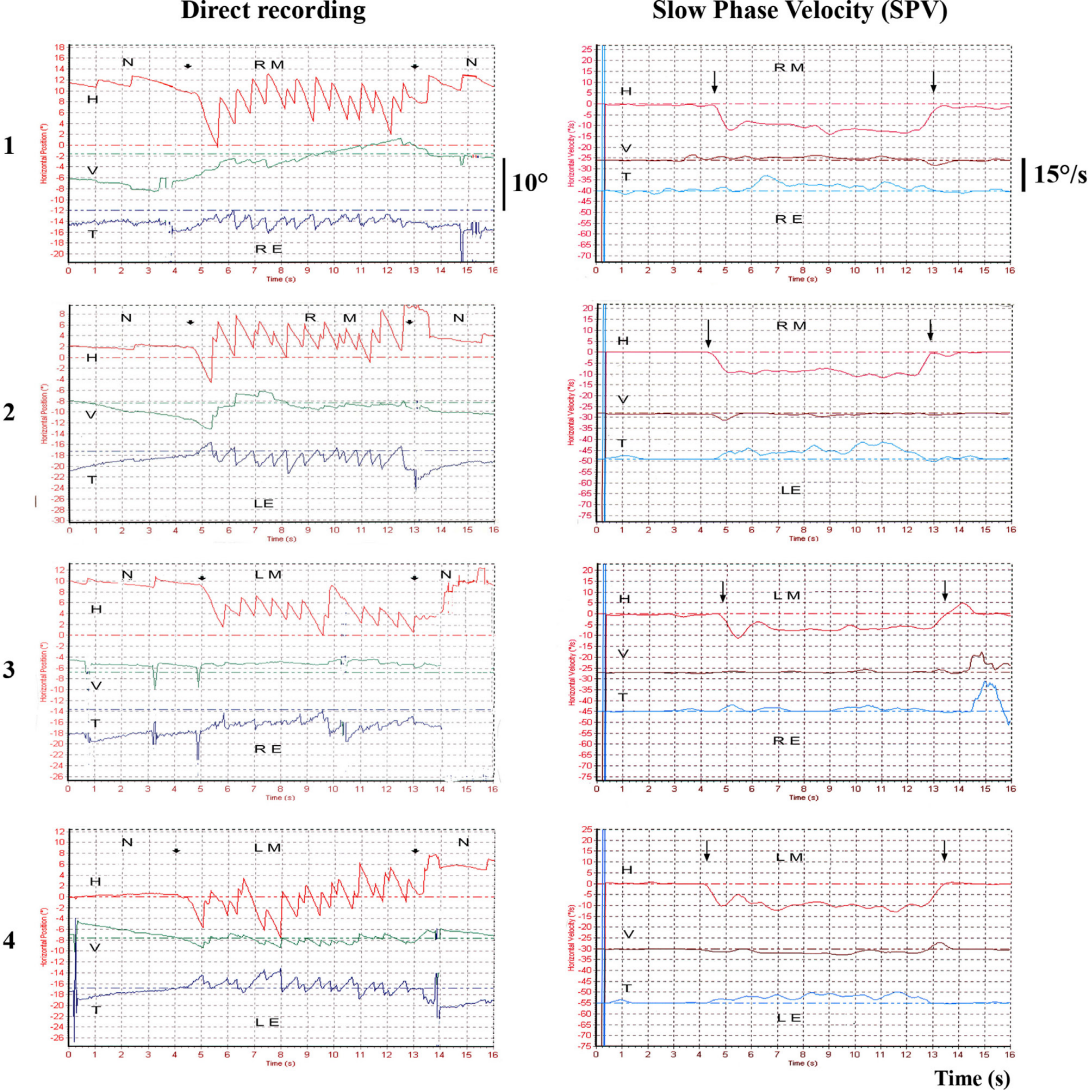
**Example of 3D recordings in a total unilateral vestibular lesion—vibration-induced nystagmus (VIN) onset and offset**. Left total unilateral vestibular loss 3D recording (translabyrinthine surgery performed 10 years ago) for vestibular schwannoma. The recordings are successively performed on the right mastoid (RM), the left mastoid (LM) with a camera on the right eye (RE) or the left eye (LE). The VIN is repeatable, reproducible on both mastoids in the same direction, beats away from the lesion side, starts and stops with the stimulation and presents no secondary reversal. H, horizontal component; V, vertical component; T, torsional component; N, no stimulus.

In tUVL patients, 100 Hz BCV applied to either mastoid induces a low velocity (~10°/s) predominantly horizontal nystagmus beating away from the affected side, irrespective of which mastoid is stimulated (3–5, 9, 10, 13, 26) (Figure 3). The nystagmus is precisely stimulus-locked: it starts with stimulation onset and stops at stimulation offset, with no post-stimulation reversal and is reproducible.

### SVIN Habituation or Fatigue after Long Period of Stimulation in tUVL

Three-dimensional recordings of 100-Hz long duration SVIN in tUVL patients during stimulation lasting 3 min demonstrated that the SVIN horizontal component persisted during stimulation with little SPV decrease, whereas first the vertical and then torsional components disappeared. These results suggest a moderate per-stimulatory adaptation (28). The brief repeated stimulation in the usual test does not show signs of habituation.

### SVIN Acts as a Vestibular Weber Test

**Figure 4:**
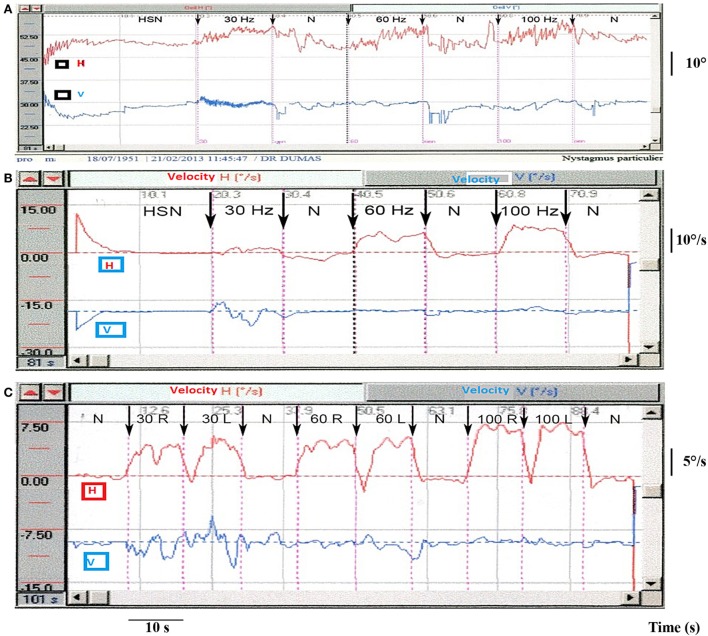
**Partial unilateral vestibular lesions [vibration-induced nystagmus (VIN) 2D recording]**. **(A)** Example of right vestibular neuritis or acute peripheral vestibular disorder (APVD). Direct recording of head-shaking-nystagmus (HSN) and VIN at 30, 60, and 100 Hz. When skull vibration-induced nystagmus test (SVINT) is performed after the HST, it is recommended to observe an interval between the two tests (about 2 min) to avoid interference of HSN on VIN due to a possible second HSN reversal phase. **(B)** Same patient, right APVD: recording of the eye slow-phase velocity (SPV). **(C)** Right chemical labyrinthectomy (intratympanic gentamicin): 2D recording of the VIN SPV; SVINT 30 Hz [right mastoid (RM)–left mastoid (LM)]; 60 Hz (RM–LM); 100 Hz (RM–LM) protocol.

In tUVL, for 100 Hz BCV mastoid stimulation, SVIN is observed in 98% of patients and beats toward the healthy side in 100% of cases (26). In addition, there was no correlation between the SVIN SPV value and which side was stimulated—either mastoid was equally effective in generating the SVIN (*P* = 0.17; *n* = 20). These results indicate that SVIN is due to stimulation of vestibular receptors on the intact side (26), and in this way, SVIN is a “vestibular Weber test” (5, 10, 27) (Figure 3). In accord with that description: in total bilateral vestibular lesions (26) and in symmetrical partial bilateral vestibular lesions, no SVIN was observed (27). Similar results are observed in severe pUVL as showed in Figure 4.

### Arguments for SVIN Being a Global Vestibular Stimulus

Three-dimensional eye movement recordings show that the response to SVIN is not purely horizontal (Figure 3). In 43 tUVL patients (10, 26) 100 Hz mastoid stimulation, the 3D recordings revealed a SVIN with horizontal, torsional, and vertical component in 98, 75, and 47% of cases, respectively. These observations suggest a primary participation of the horizontal SCC and/or utricle (for generating the horizontal component), of the posterior or superior SCC and/or sacculus (for the vertical component) and superior and posterior SCC and/or otolithic structures (for the torsional component). In SCD (7), the SVIN revealed a primarily torsional, a primarily horizontal, and a primarily vertical (up-beating in 80% of cases) component in 40, 30, and 30% of cases, respectively. These results suggest that the superior dehiscent SCC is not the only stimulated structure (the up-beating vertical component suggests the possible stimulation of the posterior SCC or sacculus) (7).

### SVIN Is Not Influenced by Vestibular Compensation Mechanisms

Dumas et al. observed in 98% of 131 surgical tUVL patients that 100 Hz mastoid vibration induced a SVIN. In well-compensated patients SVIN was not modified at 6 months, or 2 years, or 10 years (Figure 3) or up to 23 years post surgery (26). In another study measuring SVIN, the subjective visual vertical (SVV) and postural changes in a population of severe chronic compensated UVL patients (median: 32 ± 25 months) revealed a normalization of the SVV and of postural results but persistence in all patients of SVIN (30). Similarly, Hamann and Schuster (3, 59) observed that in a series of 14 unilateral unoperated VS, patients had a SVIN in 80% of cases (most of them had a chronic evolution and complained of no vestibular imbalance and had a compensated vestibular dysfunction). Ohki et al. (12) demonstrated that in 19, unoperated, long lasting VS and 15 long standing VN, a SVIN beating toward the intact side was observed in 60 and 70% of cases, respectively.

### SVINT Sensitivity and Specificity

**Figure 5:**
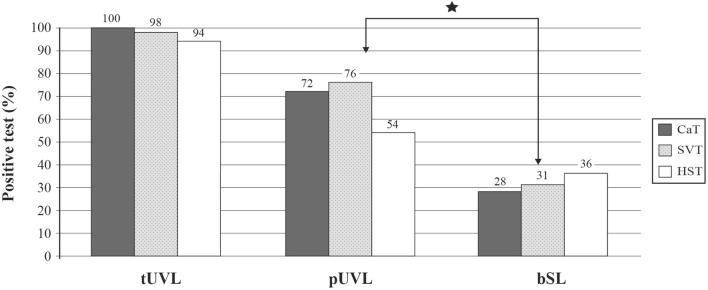
**Skull vibration-induced nystagmus test (SVINT) is more sensitive to identify peripheral than central diseases**. Comparative sensitivity of caloric test (CaT), SVINT, and head-shaking test (HST) in populations of total unilateral vestibular lesions (tUVL) (*n* = 131), of partial unilateral vestibular lesions (pUVL) (*n* = 78), and brainstem lesions (BSL) (*n* = 36). SVINT is more sensitive to reveal peripheral than central lesions (*P* = 0.04).

The sensitivity of SVIN in tUVL (*n* = 131), pUVL (*n* = 78), and brainstem lesion (*n* = 36) was 98, 75, and 30%, respectively. Specificity was 94% (*n* = 95 controls) (10, 26, 27). SVINT is significantly more sensitive for detecting peripheral disease than central brainstem lesions (BSLs) (*P* = 0.04) (10, 27) (Figure 5). The presence and direction of SVIN is strongly correlated with caloric hypofunction (26), and a SVIN is observed in 90% of UVL patients when caloric test hypofunction is higher than 50% (12).

### SVINT Is More Sensitive for Identifying Peripheral Than Central Diseases

In central or BSLs, Kheradmand and Zee (60) reported a more frequent down-beating SVIN. In BSLs, a SVIN with horizontal components beating toward the healthy side may also be observed (27) as reported by Dumas and Schmerber in cavernous hemangiomas (61). Hamann and Schuster described a SVIN in 10% of central diseases (3).

## SVIN in Various Patient Conditions

Table 1 summarizes the clinical evidence of cervical and BCVs on nystagmus. The selection criteria of publications in this comprehensive table are based on the following process.

*Data sources*: the following search criteria were used from inception through June 2016 in PubMed, Embase, Cochrane Library for key words: Skull/Head Vibrations, Vibration-Induced Nystagmus, Bone conducted vibrations, cranial vibrations, cervical vibrations, consequences on posture, vestibule-ocular-reflex (VOR) and subjective visual vertical (SVV), high frequencies, low frequencies, and nystagmus.
*Data selection*: in total, 1,723 articles were retrieved among which 1,222 satisfied to eligible criteria: vestibular structures involved, reproducibility, stimulus frequencies and location optimization, VIN characteristics, comparison with results in caloric test (CaT), ocular vestibular evoked myogenic potential (oVEMP), cervical evoked myogenic potentials (cVEMP), and first level examination tests.
*Data extraction*: critical appraisal and direct interest retained 29 clinical studies. Were retained series with sufficient number of participants, satisfactory directness of evidence (level 2 or 3), and low risk of bias.

The SVINT reveals instantaneously in UVL patients a vibration-induced nystagmus (SVIN) and so meets the need in clinical practice for a rapid, easy to perform, first-line examination test, completing the battery of common clinical tests exploring low or middle range vestibular frequencies by using higher frequency stimulations at 100 Hz. This test provides clinical guidance for further explorations or imaging as a mild, non-invasive bedside examination test with a valuable cost/efficiency rate (47, 60, 62, 63).

In summary, in unilateral peripheral vestibular lesions, SVIN is of the lesional type and beats usually toward the intact side. However, there are exceptions of this rule in partial vestibular lesions related to a combination of responses of the intact side and the residual responses of the lesion side. We report below various clinical situations highlighting these exceptions.

### Unilateral Vestibular Loss (UVL)

Two main goals of SVIN are to indicate the symmetry of the two labyrinths and lesion lateralization. Whereas the evidence is clear that SVIN indicates asymmetry, care must be taken in inferring lateralization of which side is the affected labyrinth.

In tUVL, results are simple, permanent, and consistent: whatever the stimulus frequency (40–150 Hz) and location on the skull (mastoid or vertex) the quick phase of the resulting SVIN (horizontal and torsional components) beats away from the affected ear and toward the intact side in 98% of cases (26). Karlberg et al. independently reported similar results after vestibular neurectomy (13). Results are less clear-cut in pUVL.

#### Total Unilateral Vestibular Loss (tUVL)

This condition is observed after surgical cases (translabyrinthine approaches, vestibular neurectomy) (Figure 3) or temporal bone fractures.

In tUVL, a SVIN observed in 98% of patients was always beating toward the intact side (100% of cases), and the SVIN SPV horizontal component was correlated with the total caloric efficiency on the intact side (*P* = 0.03; *n* = 20) (26). These results suggest a predominantly horizontal SCC contribution to SVIN (26) since the caloric test stimulates primarily the horizontal SCC.

In cases of tUVL, the axis of eye rotation (i.e., the relative magnitudes of horizontal, torsional, vertical eye velocity) may change with the stimulus location (mastoid or vertex) but the quick phase direction (right or left) remains unchanged with different locations (26). These results correlate closely with those obtained by concomitant caloric and head-shaking test results (10, 26).

With less complete unilateral vestibular loss (pUVL), the results are not as clear cut.

#### Partial Unilateral Vestibular Loss (pUVL)

A SVIN is observed in 75% of cases. This condition is observed in vestibular neuritis (VN), Menière’s disease, preoperative VSs, and intratympanic gentamicin (ITG).The nystagmus direction beats toward the healthy side in 91% of those cases (27). In pUVL, SVIN was significantly more frequently observed (90% of cases) when caloric testing revealed a hypofunction higher than 50% (10, 12, 27). Hamann and Schuster suggested that SVIN stimulated the horizontal SCC since they observed a SVIN correlated with the caloric hypofunction but not with cVEMP or SVV results (3, 10).

In pUVL explored with caloric (low frequency test at 0.003 Hz), HST (midrange frequency at 2 Hz), and SVINT (high frequency), it was demonstrated that the three tests were not always positive at the same time. A SVIN was observed in 20% of patients with normal calorics. Conversely caloric was positive in 22% of patients with normal SVINT. This was noteworthy in Meniere’s Disease (MD) and VS (27). In MD, nystagmus direction observed at 100 Hz with SVINT may be different from head-shaking-nystagmus (HSN) direction and be associated with a normal caloric test performed on the same day (10, 27, 60, 64). Responses may be different dependent on stimulus frequency (SVIN direction at 30 Hz is different at 100 Hz in 10% of pUVL), and discordant results between caloric test, HST, SVINT are observed in 30% of pUVL patients (27).

There are other exceptions: reports of 100 Hz SVIN causing nystagmus beating toward, rather than away from, the affected ear; in 15.5% Meniere’s Disease patients tested, 10% of VN, and 8% of pre-surgery VS cases (27). Similar exceptions have been described by Modugno et al. (15), Karkas et al. (16), Hamann and Schuster (3), Freyss et al. (17), and Negrevergne et al. (18).

### Superior Semicircular Canal Dehiscence (SCD)

**Figure 6:**
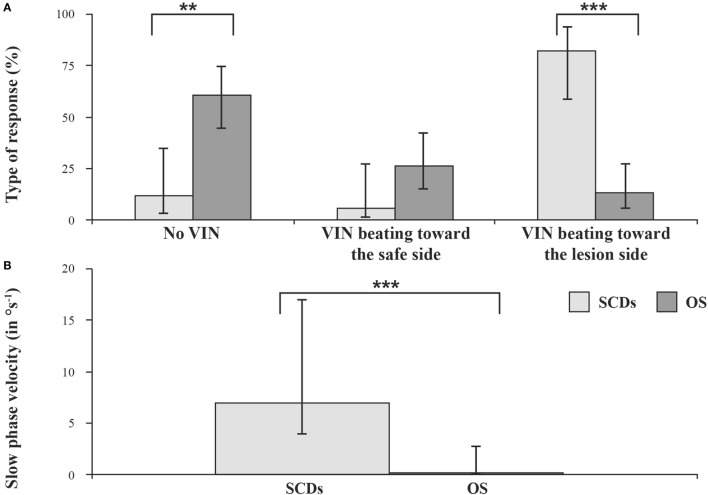
**Results in conductive hearing loss observed in unilateral SCD and otosclerosis (OS)**. Vibration-induced nystagmus (VIN) acts as a vestibular Weber Test. Skull vibration-induced nystagmus test (SVINT)—percentages (with 95% confidence interval) of no VIN, VIN beating toward the healthy side, and VIN beating toward the lesion side **(A)** and median (with interquartile range) of the slow-phase velocity of the VIN **(B)** observed in superior canal dehiscence (SCD) and otosclerosis (OS) patients (**P < 0.001, ***P < 0.0001).

In contrast to the result in UVL is the result in SCD where the nystagmus beats toward the affected ear, suggesting that the vibration activates the canal with the SCD. In unilateral SCD Dumas et al. (7) reported that the torsional and horizontal quick phases of SVIN beat usually toward the lesion side (Figure 6). In SCD, SVINT is positive in 82% of cases while caloric and HST are usually negative (7). Other authors (41–43) have also reported in SCD a prevalent stimulation of the superior SCC on the lesion side and observed a torsional SVIN beating toward the lesion side. This result is consistent with the BC facilitation inherent in this third window pathology (65–69). These results correspond to the acoumetry (Weber test) and the side of the conductive hearing loss as described by our group (7) and confirmed by Park et al. (25).

A SVIN is observed in between 82 and 100% of SCD patients (7, 41). Many authors agree that the stimulation of patients with a dehiscent anterior canal provokes a SVIN with torsional component beating toward the lesion side (6, 7, 41–43). This is opposite to the usual direction after UVL in which the unaffected ear shows the greater response. The observation of a SVIN horizontal component beating toward the lesion side is probably correlated with the concomitant stimulation of the ipsilateral horizontal SCC and/or utricle (25, 35) (Figure 6). The vertical most often up-beating SVIN suggests a more global stimulation than the sole anterior SCC (7). This pathology is associated with an increase sensitivity of inner ear structures to high frequencies. Our group described in SCD patients a SVIN observed up to 500 or 700 Hz stimulation.

### Vestibular Neuritis (VN)

Park et al. described SVIN in 63% of cases (*n* = 38) (23); Nuti and Mandala observed a SVIN in 75% (*n* = 28), a caloric hypofunction in 93%, and positive HIT in 64% of cases (21). Dumas et al. observed SVIN in 90% and a caloric hypofunction in 100% (*n* = 18) (29); Karlberg et al. described in 100% of VN an ipsi lesional tonic shift of torsional eye position (13). SVIN is most often beating toward the intact side (Figures 4A,B) but a SVIN beating toward the lesion side has been described in 10% of cases (6, 27). To summarize, a SVIN is observed in 63–100% of cases in VN usually beating toward the intact side (13, 21, 23, 27, 29) (Figure 4).

### Vestibular Schwannoma (VS)

In unoperated VS, SVIN has been described in 44–78% of cases (15, 16).

Modugno et al. (15) observed a SVIN in 44% of his 86 cases of VS beating toward the lesion side in 26% of cases; Freyss et al. (17) observed SVIN in 65% of 51 preoperative patients while the caloric (unilateral separate ear infusion) demonstrated an hypofunction in 72% of cases and a significant vestibular asymmetry after simultaneous bilateral irrigation in 95% of cases. The SVIN beats toward the lesioned side in 6% of cases. Dumas et al. reports a SVIN in 64%, a positive HST in 40%, and caloric hypofunction in 75% of 25 VS (29). In that series, the test positivity depends on the VS size (SVIN is observed in only 45% of stages 1 and 2). In another study of 70 patients, a SVIN was reported in 78% while HST was positive in 51% and caloric in 83% of cases (16). A SVIN beating toward the lesion side is observed in 10% of cases (16). Hamann identifies in 15 unoperated VS (59) a SVIN in 80% of patients with unilateral lesion and notes its absence in the only case with bilateral VS. This author does not describe precisely the nystagmus direction but suggests in most cases a nystagmus beating away from the affected ear (59).

Negrevergne et al. (18) reported in 100 unoperated VS that the results of SVIN and caloric were not always positive at the same time. SVINT was positive in 72% of cases, but a SVIN beating toward the intact side or toward the lesion side was observed in 49% and in 23% of cases, respectively.

### Meniere’s Disease (MD)

Hong et al. (22) analyzed 52 MD (between attacks) and observed a SVIN in 71% of cases beating toward the lesion side in 27% of cases. The SVIN observation was correlated with the severity of caloric hypofunction. Dumas et al. reported a SVIN in 71% of MD (most of them observed in a pre-attack or a period close to a recent attack) with caloric test results modified in 64% (29).This same author described an “irritative” SVIN beating toward the lesion side in 15.5% of cases and a frequent discordance with other tests such as caloric test or HST in 30% of cases (27). Lee (45) observed a SVIN more often in the irritative phase (63% of cases) and more rarely in quiescent periods (28% of cases). SVIN and HSN directions are discordant in 38% of MD during the irritative phase. To summarize, SVIN is observed in 28–71% of MD patients cases (usually related to the proximity of an acute period) and is often of the irritative type (22, 29).

### SVIN after Intratympanic Gentamicin (ITG)

Junet et al. (70) observed an SVIN to 100 Hz BCV in 100% of patients treated for disabling MD by ITG after seven injections with the nystagmus beating toward the intact side. After one injection, 75% showed such an SVIN. Accordingly the strength of SVIN is a guide to the severity of the deafferentation (Figure 4C). After efficient ITG in responding patients SVIN direction is correlated and concordant with the lesion nystagmus obtained in other vestibular tests and the caloric test hypofunction.

### Otosclerosis (OS)

A SVIN of low intensity is seldom observed in otosclerosis and is as often directed toward the intact as toward the lesioned side (7) (Figure 6).

### Benign Positional Paroxystic Vertigo (BPPV)

In BPPV, SVINT is seldom observed (3) and is positive only in Lindsay–Hemenway syndrome (BPPV associated with a strong ipsilateral caloric hypofunction) (10).

## SVIN Clinical Value

### A Complement to Other Vestibular Tests

Skull vibration-induced nystagmus can be conducted where caloric tests cannot, for example, where there are middle ear malformations or tympanic membrane perforations or external acoustic meatus atresia. It is useful when caloric test results are modified after middle ear surgery (radical mastoidectomy or tympanoplasty) and show a false vestibular hyperexitability (due to thermic conduction modifications). In such cases, it can substitute for the water caloric test and give informative data. This test is less invasive and challenging for elderly, arthritic, and vascular patients than HST or HIT. In conductive hearing loss with normal tympanic membrane, it can suggest an SCD if it induces a characteristic SVIN beating toward the lesion side and still observable at high frequency stimulations. This diagnosis can be confirmed by audiometric low frequency air-bone gap (bone-conducted facilitation) for the affected side related to the existence of a third window in this pathology, the stapedial reflex preservation, and further by a dedicated (targeted) imaging. In all other peripheral pathologies associated with a vestibular hypofunction, the SVIN usually beats toward the intact side. Thus, SVIN test (SVINT) provides useful information and suggests a possible hypofunctioning side (10, 27, 29).

Skull vibration-induced nystagmus test is useful for revealing false bilateral areflexia: such patients have no responses for caloric test and rotatory test (for low frequencies), no HSN, and no responses to vHIT for the six SCC (middle range frequencies) but show a SVIN proving that residual sensory hair cells at least on one side are still present and responding at high frequencies. Hence, a patient with a so-called bilateral vestibular areflexia should be documented not only with the caloric test, vHIT, and otolithic tests but also with SVIN (27).

Kheradmand and Zee (60) and Huh and Kim (62) mentioned SVIN as a part of other first-line examination tests in clinical practice and describe a nystagmus in UVL with the quick phase usually beating away from the paretic ear. Dumas et al. proposed SVIN since 1997 and 2000 (4, 5, 9), insisted on SVINT benefit as a simple test to reveal vestibular asymmetry and described the characteristics of the SVIN which was later designed as a suitable bedside clinical test and an adjunct to the caloric test (4, 5, 10, 12, 14, 24, 26–30, 47). This test has been proposed in occupational medicine (71). In clinical practice, SVINT may be part of a first-line bedside examination screening when combined with the HST, HIT (or VHIT), and possibly caloric test (or Barany test) (47, 72).

**Figure 7:**
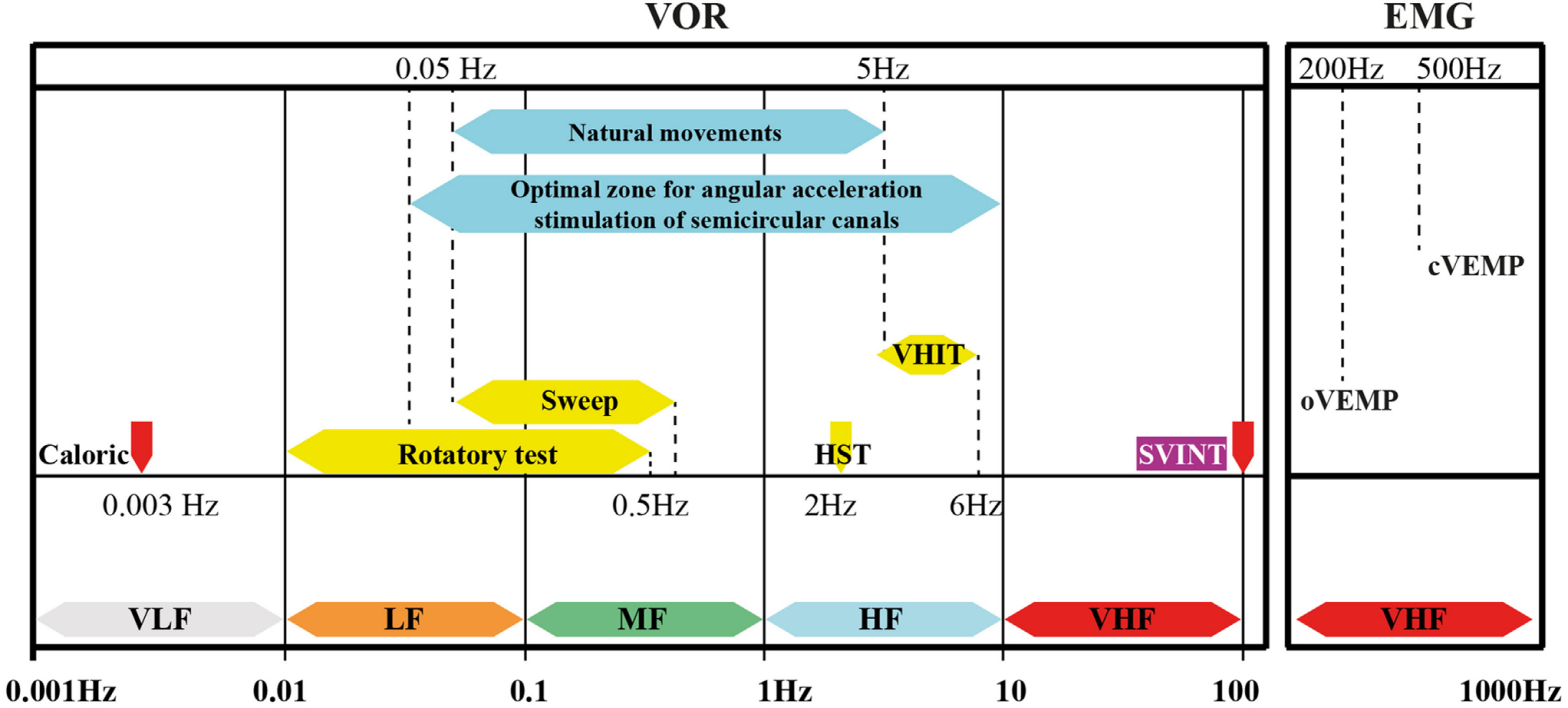
**Vibration-induced nystagmus complements other vestibular tests in the vestibule multifrequency analysis**. Place of the SVINT in the currently known frequency spectrum of the vestibular system. This graph summarizes the complementarity of vestibular tests, introduces the concept of the optimal vestibular compensation zone for the horizontal canal and the bone conduction stimulation frequencies necessary to obtain ocular vestibular-evoked myogenic potentials and cervical evoked myogenic potentials (cVEMP) (EMG). Adapted from Chays et al. (72) modified by Dumas (university PhD thesis 2014) (73). VLF, very low frequencies; LF, low F; MF, middle F: HF, high F; VHF, very high F.

Skull vibration-induced nystagmus is a recent complementary test which allows the study of vestibular frequency spectrum at higher frequencies (10, 28, 47, 72) (Figure 7) and has extended the field of vestibular exploration which has been restricted to the low frequencies of the caloric test and rotatory test. SVINT does not replace but complements the caloric test.

### Inconvenience

In case of bilateral total or symmetrical partial lesion, SVINT is negative since it does not analyze separately each side as the caloric test (47). This test uses extraphysiological stimulations since in daily life usual stimulations are between 0.5 and 5 Hz (Figure 7) (72).

### Tolerance and Adverse Effects

The test acceptability has been validated in more than 18,500 subjects (73). Some patients with recent acute peripheral vestibular disorder described lateropulsion sensations (usually toward the intact side), while other subjects with SCD may report nausea when the test is repeated. These mild manifestations usually do not prevent continuation of the examination. One subject reported transient tinnitus.

The series of 18,500 patients reported in Dumas PhD thesis (73) noted the absence of significant side effect and signaled the advantage of this test upon HST and HIT in elderly patients with vascular problems or cervical arthritis. It is recommended to perform this test cautiously in certain situations (recently operated otosclerosis, retinal detachment, history of recent cerebral hematoma, poorly controlled anticoagulant therapy) (47).

Numerous authors using BCV do not mention any noxious effect in more restraint series (3, 11–13, 23, 24, 64).

## Cervical Vibrations

Yagi and Ohyama (32) suggested in UVL patients stimulated on posterior cervical muscles that the VIN observed is the consequence of vestibular decompensation provoked by the massive proprioceptive inputs in brain stem vestibular nuclei where cervical afferents are well represented. Vibrations at 100 Hz have been described to correspond to optimal frequencies to stimulate muscles spindles (74). This conclusion may be moderated considering the possible concomitant stimulation of labyrinthine receptors because of vibrations diffusion (46) and since one knows that in UVL patients VIN SPV measured on Mastoid is more efficient than cervical posterior vibrations (10, 26).

Strupp et al. (33) explained perceptual and oculomotor effects of neck muscle vibration in VN as ipsilateral somatosensory substitution of vestibular function.

Iwasa et al.’s (34) proposal to use vibration to determine a cervical origin in vertigo was not further confirmed. They described, in vertigo with possible cervical origin, a postural sway toward the side contralateral to the vibratory nystagmus obtained in the absence of caloric test modification. These results are difficult to interpret and not totally convincing in the perspective of the VIN after cervical stimulations as an indicator of cervical proprioceptive pathology. Popov et al. (40) demonstrated in bilateral areflexive patients that neck vibrations induce a vertical upward slow eye movement and a fast phase downward and that the propriogyral illusion is secondary to vibration-induced eye movement mediated by the cervico-ocular reflex. Kawase et al. (44) demonstrated in UVL patients (VS) that neck vibrations increase significantly SVV shift and that the presence of VIN and magnitude of SVV are correlated.

Other muscular stimulations in normal subjects either cervical or to inferior limbs have been proposed: they modify posture and head position but usually induce no nystagmus (75, 76). In UVL patients, cervical stimulations induce a VIN but not inferior limbs stimulations (30).

## The Neural Basis—Evidence from Animal Experiments

The evidence for establishing the basis of SVIN comes from recordings from single primary SCC and otolithic afferents in anesthetized guinea pigs in response to BCV using frequencies and intensities comparable to those used in the clinical testing of SVIN in human (subjects and patients)—100 Hz BCV of the skull (36–38). The predominantly horizontal component of SVIN leads to the hypothesis that it is the horizontal SCC which is activated by this stimulus.

Primary vestibular afferents with irregular resting discharge were activated during low-frequency vibration of the stereotaxic frame by a hand-held Bruel and Kjaer minishaker 4810. They were identified as canal neurons by their response to angular acceleration in canal planes or as otolith neurons by their response to static pitch stimulation, and/or by neurobiotin staining. A triaxial linear accelerometer on the skull showed that the strength of the BCV stimulation in these studies was similar to that used to generate SVIN in human patients when BCV is delivered to the mastoid by a hand-held massager or dedicated device (3–5, 26, 27).

Many primary otolithic afferent neurons from the utricular or saccular macule with irregular resting discharge can be activated at low intensity by a full range of BCV frequencies from less than 100 Hz up to 2,000 Hz, with a very low threshold of about 0.02 g peak-to-peak at 500 Hz (38). When activated, the cells show phase-locking of the action potential to individual cycles of the stimulus waveform, similar to that found in auditory afferents (77). At high frequencies, the neurons do not fire on every cycle but each action potential is phase-locked to approximately the same phase angle of the vibration stimulus, so every single cycle of the waveform is the effective stimulus for the vestibular receptor/afferent.

**Figure 8:**
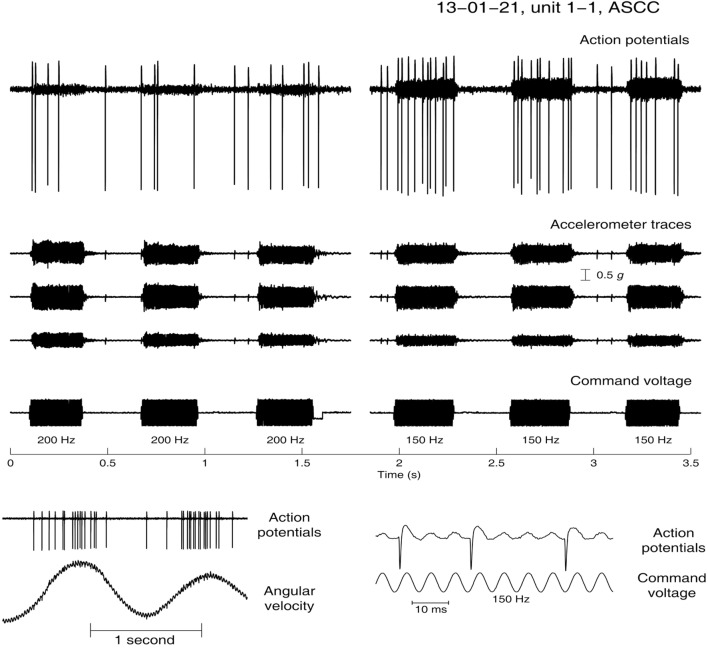
**Angular velocity data and the response to low-frequency bone-conducted vibration for an anterior semicircular canal unit**. (Bottom panel) Neural activation by angular acceleration, identifying the afferent is a canal neuron. (Top Panel) Response of the same unit to bone-conducted vibration at 200 (left) and 150 Hz (right). As stimulus frequency is decreased the neuron shows increased firing—at 200 Hz there is a modest response during the stimulus but at 150 Hz there is a strong increase in firing tightly locked to the onset and offset of the brief stimulus.

In contrast irregular horizontal SCC neurons are not activated even by high intensity (>2 g p-p) 500 Hz BCV (78). However, as the frequency is decreased to around 100 Hz, these irregular semicircular canal neurons from both the horizontal and anterior canals show activation with phase-locked firing to 100 Hz vibration and up to about 200–300 Hz (Figure 8), but no activation at higher frequencies.

This activation of SCC neurons at 100 Hz occurs even though the stimulus is a linear not an angular acceleration, and the frequency is above the highest reported upper frequency response of canal-cupula mechanical models of the horizontal canal (79). With phase locking, the 100 Hz stimulus frequency puts a limit of 100 spikes/s on the firing rate. This is above the average neural resting discharge rate of irregular semicircular canal afferents in primates (80), and such an increase in firing would also be produced by a real maintained small angular acceleration and so one would expect a horizontal eye movement response (horizontal nystagmus) to such an increased firing rate in alert animals (81).

At 500 Hz, BCV otolith neurons are clearly activated at low threshold and high sensitivity whereas semicircular canal neurons show no change in firing rate to intense 500 Hz stimuli (37, 38). This dissociation is the reason that 500 Hz BCV is used in specific clinical tests of otolith function in human patients (63, 82). However, at low frequencies around 100–200 Hz, and at stimulus levels used clinically, both otolith and semicircular canal neurons are activated. If we could have delivered even higher amplitude linear accelerations at 500 Hz, we may well have activated canal neurons but such very large linear accelerations are above the values used clinically and are impractical and painful for human subjects and patients and recording neurons would be beyond challenging.

### How Could These Results Explain the SVIN Results?

Vibration is very efficiently transmitted through the head of both guinea pigs and humans (78). As a result, the 100 Hz vibration stimulus applied to one mastoid is an effective stimulus for vestibular receptors in both labyrinths and so would be expected to cause phase-locked activation in irregular afferents from both labyrinths. Presumably healthy subjects with both labyrinths intact do not show SVIN since both labyrinths will be activated and an enhanced firing rate, phase-locked to the 100 Hz stimulus, will occur in both vestibular nerves simultaneously and so their effects on generating horizontal eye movements presumably cancel at the level of the vestibular nuclei. In patients with UVL, the irregular horizontal canal neurons on the remaining healthy side will be activated by vibration of either mastoid, and so the increased neural firing in afferents from that healthy labyrinth will not be canceled and will result in a drive to generate a predominantly horizontal nystagmus, with quick phases directed away from the affected side. Simultaneous activation of nerves from both lateral canals in cats results in cancelation of canal-induced eye movements (83).

Anterior canal neurons are also activated by the same 100 Hz (and probably posterior canals also). In other words, it is likely that all canals in a labyrinth are activated by the imposed 100 Hz mastoid BCV. So why is the nystagmus direction horizontal? The cancelation principle implies that the eye movements induced by simultaneous anterior and posterior canal activation in the one labyrinth are opposite, and so cancel, leaving just the horizontal component driving the eye movement response.

The neural results predict the following

(1) in patients with a left UVL, the 100 Hz BCV stimulation will activate irregular neurons in the healthy right labyrinth, which will not be canceled by input from the affected left labyrinth. The result will be a slow-phase eye movement to the patient’s left and quick phases to the patient’s right, i.e., a horizontal nystagmus beating away from the affected (left) side.
(2) in patients with a dehiscence of the anterior semicircular canal in the left labyrinth—the BCV stimulation will strongly activate the irregular canal afferents from the left side and not be canceled at the vestibular nuclei by the input from the right side. This will cause a horizontal nystagmus with slow phases to the patient’s right and quick phases to the patients left [i.e., a horizontal nystagmus beating toward the affected (left) side]. Moreover, in artificially created SCD, the labyrinth becomes more sensitive to high frequency stimulations for both otolith and semicircular canal receptors (84).

### Onset and Offset

In human patients, the nystagmus starts abruptly at the onset of the stimulus and finishes abruptly at the termination of the stimulus—there is no after nystagmus (4, 5, 26, 29) (Figure 3). The recordings of phase-locked activation in irregular canal and otolith neurons shows precisely the same abrupt onset and termination of phase-locked neural activation, since the mechanism of this vibration-induced activation does not involve canal-cupula mechanics (35).

The absence of any evidence of velocity storage may be due to the simultaneous otolithic activation “dumping” any canal-induced nystagmus (“tilt dumping”).

The response of single semicircular canal neurons to low-frequency BCV stimulation appears to explain the major phenomena of SVIN (Box 2). Curthoys et al. (78) demonstrated in guinea pigs that at 75 dB and 500 Hz (delivered by bone-anchored vibrators) only irregular afferent fibers issuing from otolithic vestibular structures responded; there were no responses at these frequencies issuing from SCC afferents.

Box 2 **Summary.**

1. *Why SVIN occurs in patients with asymmetric vestibular function but not in healthy subjects*: 100 Hz BCV activates semicircular canal neurons in intact (normally encased) labyrinths, and in healthy subjects the simultaneous neural input from both labyrinths would be expected to cancel, whereas in unilateral patients the input from the healthy side is unopposed so cancelation does not occur.
2. *Why the nystagmus direction is horizontal in UVL patients*: the vibration probably activates sensitive canal neurons in all canals, in both labyrinths, but the anterior and posterior canal inputs in each labyrinth will cancel, leaving just the horizontal canal activation driving the eye movement.
3. *Why 100 Hz BCV mastoid stimulation of patients with UVL causes nystagmus with quick phases beating away from the affected side*: the unopposed neural drive from the intact labyrinth will cause slow phases away from the healthy side and quick phases toward the healthy side (i.e., away from the affected side).
4. *Why 100 Hz BCV mastoid stimulation of patients with SCD causes nystagmus with quick phases beating toward the affected side*: the neural drive from the side with the SCD will have lower threshold and higher firing rate and so will not be fully canceled by the activation from the healthy ear resulting in slow phases away from the affected side and quick phases toward the affected side.
5. *Why these respective responses in patients are independent of which mastoid is stimulated*: the vibration stimulation is conducted so effectively to both labyrinths, independently of which side is stimulated.
6. *Why the onset and offset of the nystagmus is so abrupt, unlike other nystagmus attributed to horizontal canal activation by angular acceleration*: the vibration stimulus causes immediate phase-locked activation of canal neurons which ceases at stimulus offset.
7. *Why high frequency vibrations at 100 Hz which are beyond the mechanical cut off frequencies of canal mechanism*: BCV induces inner ear fluid displacements which deflect type I receptors and so activates irregular afferent neurons.

In a more recent work (36), the authors studied in guinea pigs responses to a wider range of frequencies and demonstrated that at 100 Hz in normally encased labyrinths both canal and otolithic irregular fibers were activated. Lower stimulus intensity (in g) is, however, required to elicit responses from otolithic structures than from canals. For frequencies at 500 Hz or higher, only otolithic afferents from the striola region of utricular or saccular macula were activated (36, 78).

Vibration-induced nystagmus requires not only integrity of the peripheral end organ (type 1 inner ear hair cells), afferent neurons with irregular discharges, and integration in the vestibular nucleus for production of the slow phase but also structures of the brain stem to restore the eye position by quick phases (pontine reticula formation). Clinical interpretation of VIN in term of topography and side of a lesion needs a careful analysis and we emphasize that although SVIN is robust and the 100-Hz mastoid vibration stimulus is superficially simple, care must be taken with patient testing, stimulus presentation, response measurement, and the interpretation of the results (Box 3).

Box 3 **SVIN validity criteria.**

The induced nystagmus starts with stimulation, stops with its withdrawal, and does not present any secondary reversal. It is sustained, reproducible, and beats in the same direction either after left and/or right mastoid (RM) process stimulation, is often less intense or absent after vertex stimulation (except in case of superior SCD). Nystagmus is usually absent or discordant in subjects with no vestibular disorders: in 10% of cases, false-positive nystagmus is observed at 60 Hz with right-beating nystagmus on the RM and left-beating nystagmus on the left mastoid (inconsistent directions). A SVIN slow-phase velocity SPV greater than 2°/s is also required to validate the test.

## Conclusion

Skull vibration-induced nystagmus test, a recent, robust, non-invasive examination test, has opened a new area of vestibular exploration as it allows without side effects a simple non-invasive, rapid clinical test to vestibular high frequencies. SVINT is a useful tool to indicate a lesion side and reveals instantaneously, even in chronic or compensated patients, a SVIN in case of vestibular asymmetry as a vestibular Weber test. SVINT is a reliable, fast, first-line test. The optimal frequency to induce a SVIN is 100 Hz.

Skull vibration-induced nystagmus test is useful to complement information of other common vestibular tests in the multifrequency analysis of vestibular function. Yet, it gives no specific information on vestibular pathways exact topographic alteration and reveals modifications related with a lesion located in any point of the vestibulo-ocular reflex pathway. Noteworthy, it is significantly more sensitive to reveal peripheral than central lesions.

Skull vibration-induced nystagmus test is not influenced by vestibular compensation and could be advisable as an additional data and an adjunct in forensic or occupational medicine. It brings complementary information to classical SCC test explorations, cVEMP, and oVEMP. Its use in clinical practice seems to predict a wider development as a future promising first-line test.

## Author Notes

The skull vibration-induced nystagmus test was the subject of the PhD thesis entitled “Influence of vibratory stimulation applied to the skull and neck muscles on equilibrium function. Physiological interpretations and applications to pathology. Development and validation of a new vestibular investigation test: the skull vibration-induced nystagmus test or Dumas test” presented at the University of Lorraine (Nancy), September 18, 2014. Thesis Jury Members: S. Caudron, France; A. Charpiot, France; N. Deggouj, Belgium; P. Denise, France; H. Kingma, The Netherlands; M. Magnusson, Sweden; P. Perrin, France; S. Schmerber, France; D. Vibert, Switzerland.

## Author Contributions

All authors listed, have made substantial, direct and intellectual contribution to the work, and approved it for publication.

## Conflict of Interest Statement

IC is an unpaid consultant to GN Otometrics. The other authors declare no conflict of interest.

## Abbreviations

BCV, bone-conducted vibration; SCC, semicircular canal; SPV, slow-phase eye velocity; SVIN, skull vibration-induced nystagmus; SVINT, skull vibration-induced nystagmus test; UVL, unilateral vestibular loss; pUVL, partial unilateral vestibular loss; tUVL, total unilateral vestibular loss; VOR, vestibulo-ocular reflex.
